# Engineering of Multifunctional Nanocomposite Membranes for Wastewater Treatment: Oil/Water Separation and Dye Degradation

**DOI:** 10.3390/membranes13100810

**Published:** 2023-09-25

**Authors:** Hamouda M Mousa, Mostafa M. Sayed, Ibrahim M. A. Mohamed, M. S. Abd El-sadek, Emad Abouel Nasr, Mohamed A. Mohamed, Mohamed Taha

**Affiliations:** 1 Mechanical Engineering Department, Faculty of Engineering, South Valley University, Qena 83523, Egypt; 2 Faculty of Technological Industry and Energy, Thebes Technological University, Thebes, Luxor 85863, Egypt; 3 Department of Mechanical Design and Materials, Faculty of Energy Engineering, Aswan University, Aswan 81542, Egypt; 4 Department of Chemistry, Faculty of Science, Sohag University, Sohag 82524, Egypt; ibrahim_mohamed@science.sohag.edu.eg; 5 Nanomaterials Lab., Physics Department, Faculty of Science, South Valley University, Qena 83523, Egypt; mahmoud.abdelsadek@sci.svu.edu.eg; 6 Physics Department, Faculty of Science, Galala University, Galala, Suez 43511, Egypt; 7 Department of Industrial Engineering, College of Engineering, King Saud University, P.O. Box 800, Riyadh 11421, Saudi Arabia; eabdelghany@ksu.edu.sa; 8 School of Engineering, University of South Wales, Pontypridd CF37 1DL, UK; mohamed.mohamed@southwales.ac.uk; 9 Mechanical Engineering Department, College of Engineering and Technology, Arab Academy for Science, Technology and Maritime Transport, Sadat Road, Aswan 81511, Egypt; mohamed_taha@aast.edu

**Keywords:** nanocomposite membrane, photocatalytic, oil/water separation, antibacterial activity

## Abstract

Multifunctional membrane technology has gained tremendous attention in wastewater treatment, including oil/water separation and photocatalytic activity. In the present study, a multifunctional composite nanofiber membrane is capable of removing dyes and separating oil from wastewater, as well as having antibacterial activity. The composite nanofiber membrane is composed of cellulose acetate (CA) filled with zinc oxide nanoparticles (ZnO NPs) in a polymer matrix and dipped into a solution of titanium dioxide nanoparticles (TiO_2_ NPs). Membrane characterization was performed using transmission electron microscopy (TEM), field emission scanning electron microscopy (FESEM), and Fourier transform infrared (FTIR), and water contact angle (WCA) studies were utilized to evaluate the introduced membranes. Results showed that membranes have adequate wettability for the separation process and antibacterial activity, which is beneficial for water disinfection from living organisms. A remarkable result of the membranes’ analysis was that methylene blue (MB) dye removal occurred through the photocatalysis process with an efficiency of ~20%. Additionally, it exhibits a high separation efficiency of 45% for removing oil from a mixture of oil–water and water flux of 20.7 L.m^−2^ h^−1^ after 1 h. The developed membranes have multifunctional properties and are expected to provide numerous merits for treating complex wastewater.

## 1. Introduction

Modern industries have resulted in various water pollutants, such as dyes and oils split apart [1]. Physical and biological treatments have high stability, and their aromatic nature renders them ineffective [2]. Moreover, physical techniques [3] have the potential to induce the transference of organic compounds to another phase, thereby leading to the emergence of secondary pollution and additional costs [4,5]. The degradation of pollutants from wastewater, particularly dyes, through photocatalysis is a highly promising, uncomplicated, and economical approach [6,7,8]. These dyes such as methylene blue (MB) are toxic and non-biodegradable and cause environmental pollution that affects human health [9]. Membrane technology plays a role in water treatment due to its easy operation, cost-efficiency, high productivity, and removal capacity [10,11]. Photocatalytic membranes (PMs) exhibit inherent antimicrobial properties, exceptional hydrophilicity, robust photocatalytic oxidation capabilities, and a distinct separation mechanism. These characteristics enhance the photocatalytic destruction of organic contaminants, bacteria, and viruses found in water. The contaminants can be effectively degraded to carbon dioxide, water, and nitrogen gas while operating under ambient temperature conditions. Moreover, an additional benefit of employing photocatalytic membranes is the requirement to isolate nano-photocatalyst materials from the system. This particular aspect will encourage the utilization of photocatalytic membranes in water treatment [12,13]. Furthermore, photocatalytic membranes can potentially serve as a pioneering solution for oil–water separation processes. Nanomaterials in ultra-hydrophilic membranes have advanced oily effluent separation processes. Polymeric membranes prevent oil droplets from adhering and reducing filtering resistance [14]. This is due to their ability to effectively mitigate fouling issues and facilitate chemical and energy free cleaning through solar light irradiation. A cellulose-based membrane has capabilities for oil–water separation and wastewater purification treatments. There are several applications of cellulose-based membranes in photocatalytic degradation, desalination and purification, and reverse osmosis [15]. 

Photocatalytic nanomaterials offer advantages for preparing highly hydrophilic, self-cleaning membranes, as they can decompose organic pollutants from the surface and organic contaminants of fouled pores without secondary pollutants [16,17]. Metallic oxides, including zinc oxide (ZnO) and titanium dioxide (TiO_2_), exhibit significant photocatalytic activity when exposed to sunlight. For example, ZnO has been used as a photocatalytic material due to its bandgap energy of 3.2 to 3.3 (eV), which makes it a promising photocatalyst material for hydrogen production [18]. Electrical topologies that reduce the likelihood of electron–hole recombination and promote effective charge transit dynamics are attributed to p-n heterojunctions. One significant observation is that semiconductors with the same photocatalytic properties are incapable of capturing photons within the visible light spectrum, as they only harvest approximately 4% of the total solar spectrum, as reported by Revathi [19] and Panthi [20]. Notably, nearly all materials of this nature experience photocarrier recombination, resulting in a decrease in the efficacy of hydrogen evolution [19]. In the other hand, TiO_2_ is a highly researched photocatalytic material owing to its favorable attributes such as abundant availability, low cost, remarkable chemical stability, and notable photocatalytic activity. Notwithstanding the manifold benefits of TiO_2_, its activation predominantly relies on ultraviolet (UV) radiation, which constitutes a minor proportion of solar radiation. Consequently, the employment of pure TiO_2_ in solar systems results in restricted efficacy in the decomposition of hydrocarbons [21,22]. However, TiO_2_ exhibits a high band gap energy of 3.2 eV and rapid charge recombination (photoinduced electrons and holes), constraining its photocatalytic efficacy [23]. Efforts have been made in research to enhance efficiency by developing composites of TiO_2_ with noble metals, graphene oxide (GO), metal-organic frameworks (MOFs), carbon nanotubes (CNTs), and semiconductors [24,25,26,27]. Using nanocomposites inhibits the recombination process of electron–hole pairs generated by light, enhancing the efficiency of photocatalysis [28]. Photocatalytic reactions generate electron–hole pairs after high-energy irradiation. The combination of distinct semiconductor oxides effectively separates electrons and holes, leading to elevated levels of photocatalytic performance [29]. TiO_2_ and ZnO have been investigated as semiconductor photocatalysts to boost photocatalytic characteristics [30]. These combinations include TiO_2_/ZnO nanocomposite films, TiO_2_/ZnO polycrystalline powders, and TiO_2_/ZnO composite powders which have a good photocatalytic activity performance [31,32,33]. Accordingly, photocatalytic materials applied to solar-light active membranes can significantly enhance pollutant removal efficiency and membrane surface cleaning [34].

The present work examines the engineering of a multifunction photocatalytic membrane that can be utilized for dye degradation, oil–water separation application, and water disinfection treatment. The fabricated membrane is composed mainly from cellulose acetate (CA) as a polymer matrix and ZnO NPs immersed into TiO_2_ NPs solution as a photocatalytic substitute. The implementation of this particular strategy has the potential to significantly improve both the photodegradation and antibacterial properties of oily wastewater and dyes in industrial wastewater. In addition, using polymeric membranes with nanoparticles would serve nanoparticles for recycling [35]. Electrospinning is used to fabricate a composite membrane of CA/ZnO NPs followed by immersion in a suspension solution of TiO_2_ NPs to facilitate the deposition of TiO_2_ NPs on the surface of the nanofibers. The use of a CA/ZnO @ TiO_2_ NPs-coated membrane for oil–water separation is a proposed strategy to develop a multifunctional membrane.

## 2. Materials and Methods

### 2.1. Materials

All chemicals and other materials utilized in this study were purchased from Sigma Aldrich. These included cellulose acetate (with an average molecular weight (Mn) of approximately 50,000 and an acetyl content ranging from 39.20% to 40.20% by weight), titanium dioxide nanoparticles (TiO_2_ NPs) nanopowder, <25 nm particle size, 99.7% trace metals basis, zinc oxide nanoparticles (ZnO NPs) nanopowder, <50 nm particle size (BET), >97%, acetone, and acetic acid.

### 2.2. Membrane Fabrication

**Figure 1:**
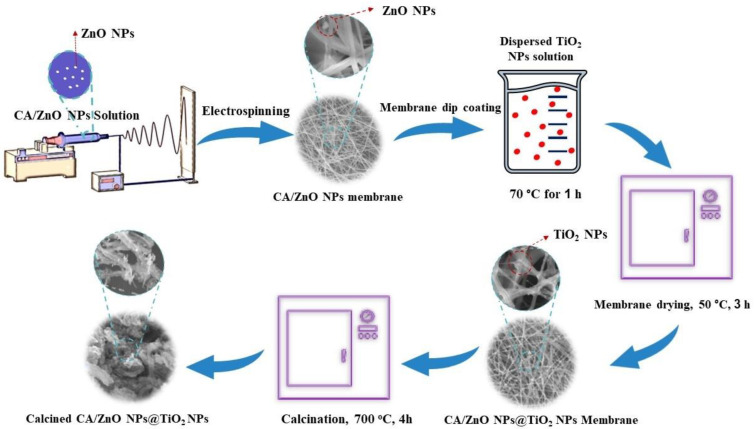
Illustrative diagram shows nanocomposite membrane fabrication steps: electrospinning setup component, dip coating of TiO_2_ NPs, membrane drying, and calcination.

Cellulose acetate (CA) powder with an 18 wt.% concentration was dissolved in a 3:1 mass ratio of acetic acid to acetone for 12 h, and the mixture was stirred at 200 rpm at room temperature. Then, 0.5 g of ZnO NPs was added to 18 wt% of the polymer solution, which used to form a composite nanofiber. The polymer solution was sonicated for 1 h after being stirred at 200 rpm overnight, which assists to better disperse the nanoparticles throughout the polymer matrix. Nanofibers were produced using the electrospinning method, as illustrated in Figure 1. Parameters such as the syringe inner diameter, feed speed, and collecting distance starting from the needle were adjusted during electrospinning, with values of 0.52 mm, 1.0 mL/h, and 170 mm being used, respectively. Alligator clips were used to link a high-voltage DC power source to the anode and an aluminum-foil-covered flat plate served as the collector and held the needle in place. A high-voltage power supply (ESN-HV30/ESN-HV30N, NanoNC, Seoul, Republic of Korea) and a syringe pump (supplier: NanoNC, Republic of Korea) were used at a high voltage of 20 kV at room temperature for the electrospinning process [36].

A sonication bath was used to disperse the TiO_2_ NPs into the distilled water, after which the developed nanocomposite CA/ZnO NPs membrane was placed in the bath. After 1 h in a sonication bath at 70 °C, the membrane was coated with TiO_2_ NPs suspended in distilled water and then dried at 50 °C for 3 h. A VULCAN 3 -550 electric furnace was used to calcine the resultant composite of (CA/ZnO NPs)/TiO_2_ NPs for 4 h at 700 °C.

### 2.3. Membrane Characterization

Field emission scanning electron microscopy (FESEM) with energy dispersive X-ray spectroscopy (FESEM-EDX; Quanta FEG 250; Japan) was applied to describe the morphologies of the introduced membranes. Creating a high-resolution image with a better conductive surface, a 10 nm gold film was applied to the small-size membranes before investigation using FESEM. Nanofibers of the composite membranes were analyzed with transmission electron microscopy (TEM), using JEOL’s JEM-2100 model. FT-IR (4100, JASCO, Tokyo, Japan) was used to evaluate the chemical bonding of blended membranes. Moreover, the water contact angle was used to test membrane wettability using the DPRO image standard (Phoenix, SEO, New York, NY, USA).

### 2.4. Antibacterial Properties

The experimental procedure for evaluating the synthesized samples’ antibacterial properties was conducted according to our previous report [14]. In brief, various materials were assessed using a strain of Gram-negative bacteria, namely E. coli. The Muller–Hinton agar plate model was utilized and subsequently incubated at 37 °C for 1–2 days while maintaining a pH of 7.3 ± 0.1. The circular filter paper, measuring 6 mm in diameter, underwent sterilization through autoclaving at a temperature of 121 °C for 20 min. The sample used for the experiment had a concentration of 1.5 × 10^8^ CFU/mL and was obtained from various sources. The discs that had been impregnated were subjected to incubation at 37 °C for 48 h. A positive control was utilized, with 30 mg/disc of Tetracycline being employed. The investigated hybrid materials and growth inhibition diameter were in millimeters (mm).

### 2.5. Measurements of Membrane Performance

The CA/ZnO @ TiO_2_ NPs membrane was placed within a magnetic filter funnel (PALL, 300 mL, Port Washington, NY, USA) to function as a separation membrane. A 20 cm^2^ area of effective filtration was measured for the funnel. The polyphenylene sulfone measuring cup contained the oil/water mixtures at a concentration of 50 *v*/*v*%. The separation was accomplished through the differential weight of the liquids. Concurrently, in order to assess the membrane separation efficacy, stability, separation efficiency, and liquid flux were measured for the various cycles of the oil removal mode.

The flow (J) of liquid across the membrane was calculated according to the following equation:

J = V/A × ∆t (1)

where A (m^2^) is the effective (surface) area of the separation membrane in the setup, V (mL) is the volume of filtrated liquid, and t (h) is the filtration time.

Equation (2) was used to determine each mixture’s separation efficiency.

ɳ = V_1_/V_0_ × 100 (2)

V_0_ is the water volume before separation, while V_1_ is the authorized water volume after separation.

Methylene blue (MB) with a concentration of (10 ppm) according to previously reported work was used as simulation wastewater [37]. Samples with dye concentrations were exposed to direct sunshine irradiation and were used as a straightforward photochemical reactor to test the photocatalytic materials. Each experiment maintained the produced photocatalytic nanomaterial’s initial concentration and mixed solution volume. A 50 mL glass beaker contained 10 mg of the manufactured components and a 15 mL organic dye solution (10 ppm). To reach desorption-absorption equilibrium before usage, the combined solution was agitated for 30 min in the dark. After that, the initial concentrations of the dye mixture were measured using a spectrophotometer. After that, samples in the beakers were placed in the sun. The efficiency of the photocatalyst material was then evaluated after samples of 1 mL were tested at various intervals, centrifuged, and measured for absorbance at a maximum wavelength of 646 nm. Regarding the UV-vis absorption peak, both MB samples had corresponding wavelengths of 646 nm. The rate of dye deterioration was calculated using the following equation:

Degradation efficiency (%) = (1 − C/C_0_) × 100% (3)

C_0_ is the initial concentration, while C is the dye concentration throughout irradiation. Members of the engineering faculty of South Valley University in Qena, Egypt, conducted all experiments on a sunny day between 2 and 4 PM in June with high-intensity solar light and 25 MJ/m^2^ solar radiation.

## 3. Results

### 3.1. Morphology and Structure Analysis

**Figure 2:**
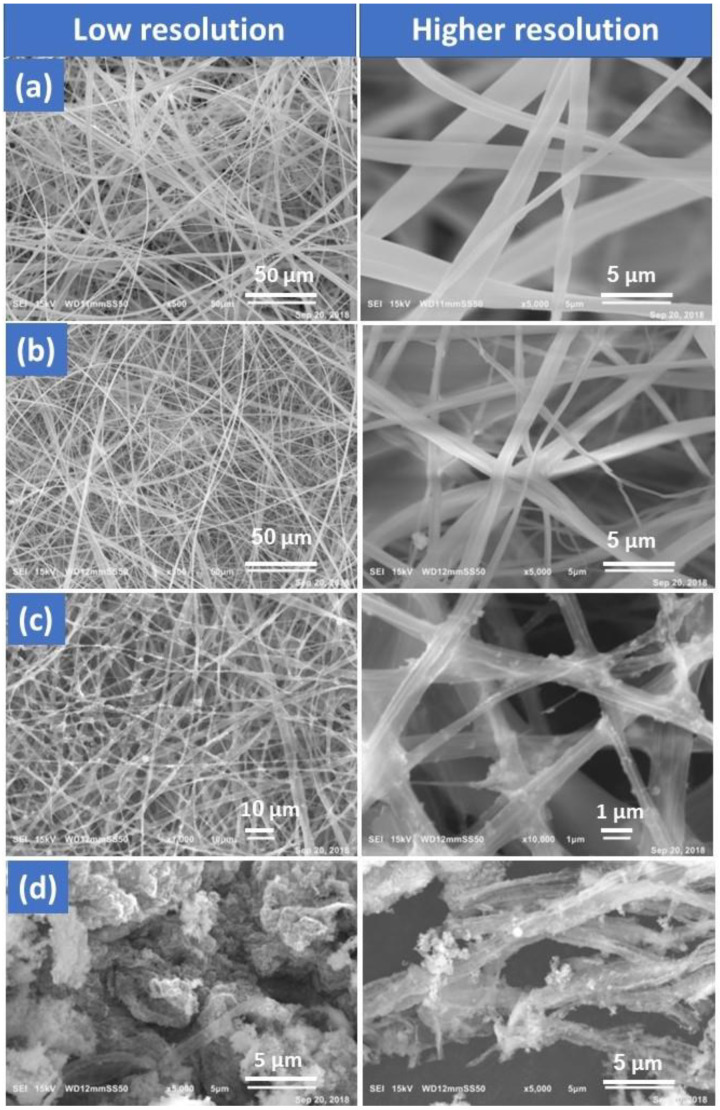
FESEM analyses show membrane morphology: (**a**) CA, (**b**) CA/ZnO NPs, (**c**) CA/ZnO @ TiO_2_ NPs, and (**d**) calcined CA/ZnO @ TiO_2_ NPs.

Inorganic chemicals are challenging to spin into fibers. As a result, viscoelastic polymers are employed as fiber-assisting templates to create inorganic/polymer hybrid precursor fibers before they are converted to inorganic fibers. Figure 2 illustrates FESEM images of the fabricated membrane morphology. Figure 2a represents an FESEM image of pure CA nanofibers exhibiting rather smooth, spherical threads that were consistently distributed. Figure 2b shows FESEM images of the nanofiber composite CA/ZnO NPs. The findings demonstrated the production of nanofibers without beads, which may be attributed to ideal circumstances and effective ZnO NP integration. The membrane nanofiber surface of the third sample (Figure 2c) was coated with a dispersed TiO_2_ NPs solution using a dip coating process in a water bath at 70 °C, followed by drying at 130 °C for 60 min. The resultant composites were imaged using FESEM, and the images showed nanoparticles forming on the nanofibers’ outermost surface. In contrast to expectations, sheet-like particles rather than nanofibers were unexpectedly produced during the calcination of CA/ZnO/TiO_2_ composite nanofibers at 700 °C in the oven (Figure 2d). The supporting CA polymer must be switched from a thermoplastic to a thermosetting polymer in order to create ZnO/TiO_2_ composite nanofibers that resemble fibers.

**Figure 3:**
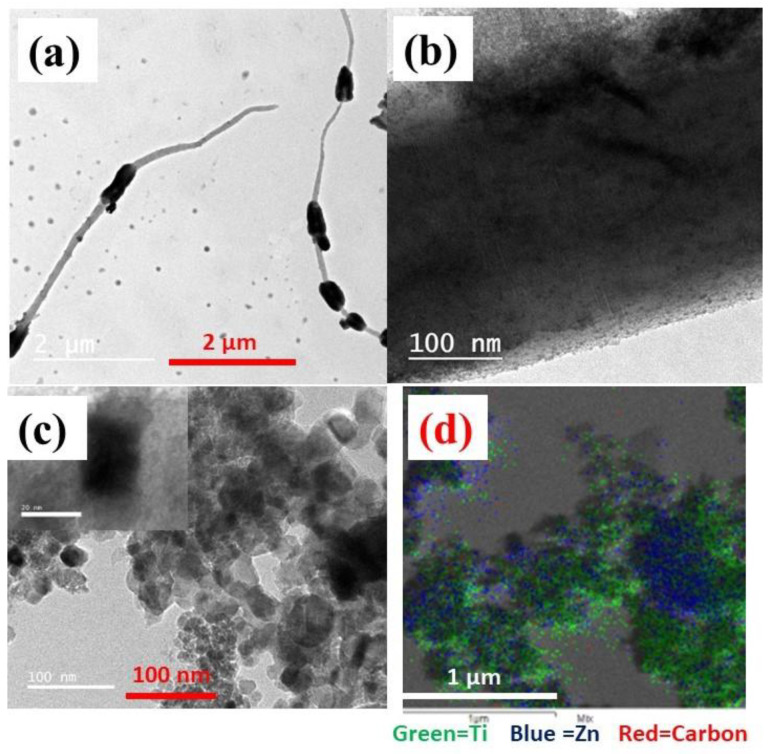
(**a**,**b**) Characterization of CA/ZnO @ TiO_2_ NPs membrane of single nanofiber, (**c**,**d**) calcined CA/ZnO @ TiO_2_ NPs showed TEM images of composite nanoparticles and EDS mapping.

The CA/ZnO @ TiO_2_ NPs nanofiber membrane and calcined CA/ZnO @ TiO_2_ NPs sample at lower and higher resolution TEM images are shown in Figure 3. The CA/ZnO @ TiO_2_ NPs nanofiber membrane exhibits integration of ZnO NPs within the nanofiber structure and the presence of TiO_2_ NPs on the membrane’s upper surface. The HR-TEM images of one fiber shown in Figure 3a,b reveal ZnO dispersion throughout the fiber, along with the crystal distribution and the attachment of TiO_2_ NPs on the membrane surface. TEM images and EDS mapping of the calcined sample confirm the existence of ZnO and TiO_2_ NPs, as indicated in Figure 3c,d. TEM was used to measure the size of nanoparticles of ZnO and TiO_2_. TiO_2_ typically appears as green spots and ZnO as blue to determine the size of each nanoparticle, which ranges from 16–72 nm and 36.7 nm, respectively. EDS mapping also displayed the elemental distribution in the CA/ZnO @ TiO_2_ NPs sample to investigate the elemental distribution.

### 3.2. Chemical Composition (FTIR)

**Figure 4:**
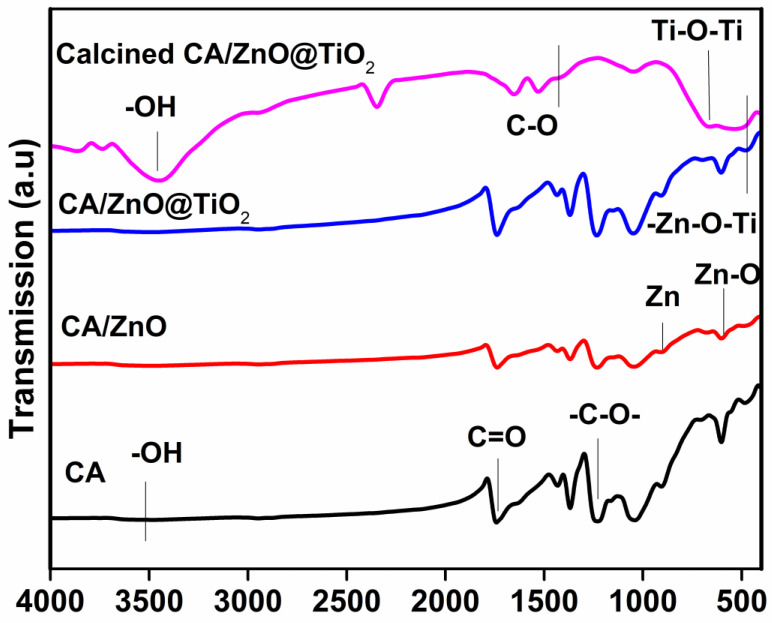
FTIR analysis of the different developed membranes’ conditions.

Figure 4 displays FTIR spectra of the fabricated membranes. The analysis of the pure CA nanofiber exhibited a distinctive absorption peak related to the carbonyl (C=O) bond stretching at wavenumbers 1752 and 1236 cm^−1^. This absorption band can be attributed to the presence of the acetate substituent, as indicated by the C-O-C alkoxyl stretching. The band at 3480 cm^−1^ was also assigned to an O-H stretching vibration, while the band at 1368 cm^−1^ was assigned to a C-CH_3_ methyl bending vibration [38]. The CA @ ZnO composite membrane exhibits a -C-O vibration, albeit with a discernible 10 cm^−1^ black shift compared to cellulose. This observation reveals a slight reduction in the strength of the C-O bonds within the cellulose, potentially resulting from a chemical interaction with ZnO. The CA@ZnO membrane shows distinct peaks at 894, and 603 cm^−1^. These peaks correspond to the tetrahedral coordination of Zn formation, and stretching vibrations of Zn-O bond, which suggest the presence of ZnO [39,40]. As reported in the literature, the absorption peak observed at 500–900 cm^−1^ is attributed to the Ti-O stretching and Ti-O-Ti bridging stretching modes in TiO_2_ [41]. The findings of this study demonstrate the successful grafting of TiO_2_ NPs onto the surface of the CA/ZnO @ TiO_2_ NPs membrane. The FTIR spectra obtained from the calcined sample show absorption peaks observed at 3440 cm^−1^ that are attributed to physically adsorbed water molecules containing (-OH) groups [42]. Additionally, the band detected at 1400 cm^−1^ can be attributed to the stretching vibration of carbon-oxygen (C-O) bonds [43]. Furthermore, the absorption peaks at 550 and 650 cm^−1^ are attributed to stretching vibrations of the Ti-O-Ti and the vibration mode of the -Zn-O-Ti groups, respectively [42]. The findings indicate successfully creating a uniform composite nanofiber membrane consisting of CA/ZnO. Additionally, the excellent deposition of TiO_2_ NPs onto the material surface of the CA/ZnO @ TiO_2_ NPs membrane was observed. Furthermore, doping the two semiconductor materials was achieved through the calcination/annealing of the sample, forming a composite S-S heterojunction material.

### 3.3. Wettability and Antibacterial Test

**Figure 5:**
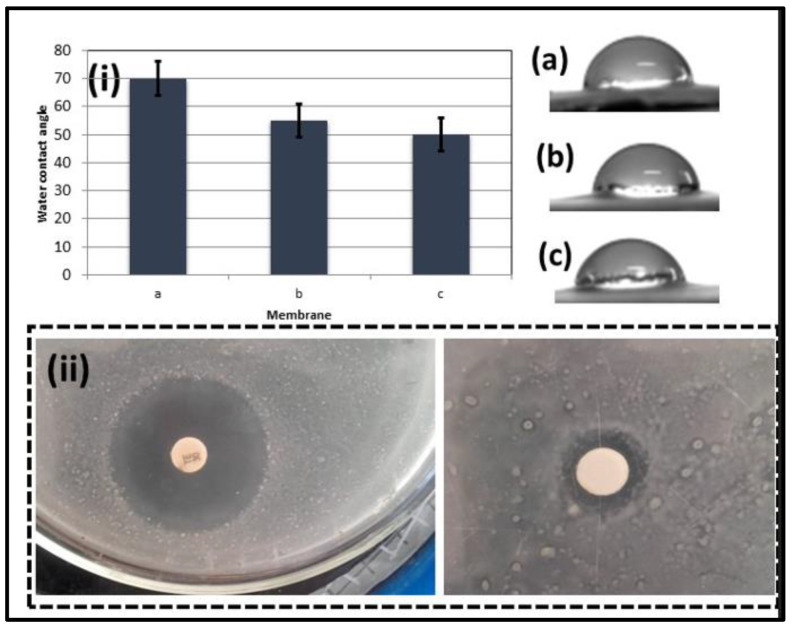
(**i**) Membrane wettability using WCA: (**a**) CA, (**b**) CA/ZnO NPs, and (**c**) CA/ZnO @ TiO_2_ NP. (**ii**) antibacterial test of the control and CA/ZnO @ TiO_2_ NPs membrane.

Water contact angles (WCAs) of the three membranes are illustrated in Figure 5i. The findings show that the pure CA membrane exhibits inferior wetting properties compared to the CA/ZnO NPs and CA/ZnO @ TiO_2_ NPs membranes. Notably, the WCA value of the CA/ZnO @ TiO_2_ NPs membrane is the lowest, recording 50°. This is due to the fact that ZnO and TiO_2_ NPs can adsorb hydroxyl groups (-OH) that are hydrophilic, enhancing the surface energy of membranes and making them more hydrophilic [44,45]. Consequently, incorporating ZnO and TiO_2_ NPs augments the surface energy, facilitating the membrane’s capacity to absorb water [46,47]. The membrane matrix’s nanoparticles act as sponge-like structures featuring interconnected pores of uniform size, resulting in enhanced water flux and improved permeability [48]. These findings indicate that the hydrophilicity of the CA/ZnO @ TiO_2_ NPs membrane is superior to that of both the pure CA membrane and the CA/ZnO NPs membrane. The observed low value of the contact angle of the CA/ZnO @ TiO_2_ NPs membrane suggests that the membrane CA/ZnO NPs coated with TiO_2_ NPs possesses promising capabilities for use in oil–water separation applications.

Wastewater contains diverse living organisms, including microbial and other contaminants. Zinc oxide (ZnO) with antibacterial activity was aimed at eliminating and disinfecting wastewater originating from living organisms. Figure 5ii presents the optical depiction of the inhibition zone of the control sample and the CA/ZnO @ TiO_2_ NPs membrane. The findings suggest that the composite membrane exhibits the most substantial inhibition zone, measuring at 15 ± 0.8 mm, compared to the control sample (Tetracycline), which measures a 30 mm inhibition zone in the Gram (-ve) bacteria test. According to the results, the synthesized sample contains about 50% of the reported antibacterial material (Tetracycline), indicating that the composite TiO_2_/ZnO NPs have generated a unique chemical structure with increased antimicrobial characteristics [14]. The primary benefits of the resultant inorganic oxide antibacterial material stem from its biocidal properties, which have been observed to induce zone inhibition and bacterial eradication, specifically in the case of Escherichia coli. The interaction of materials with the bacterial cell membrane in the presence of water elucidates the antibacterial activity mechanism, as a result of which reactive oxygen species are produced, including hydroxyl radicals, superoxide, and H_2_O_2_. The OH^•^ radicals and/or superoxide containing negatively charged particles can effectively traverse the cell membrane and persist on the external surface of bacteria, impairing proteins, lipids, and DNA. In contrast, it has been observed that H_2_O_2_ can permeate the cell membrane and effectively eliminate bacteria, thereby serving as a disinfectant agent in wastewater treatment through the photocatalytic and separation process [49,50].

### 3.4. Membranes’ Photocatalytic Activity

When a semiconductor photocatalyst surface is exposed to light photons, it undergoes heterogeneous photocatalytic reactions [51]. Electrons (e^−^) from the valence band (VB) of the nanoparticles (NPs) are excited to the conduction band (CB) when the light of photon energy is greater than the semiconductor’s bandgap energy that is irradiated. This process generates positively charged holes (h^+^) in the VB, which can either recombine, creating thermal energy that hinders the photocatalysis process, or diffuse to the surface of the photocatalyst and react with the adsorbed molecules. The holes (h^+^) react with H_2_O, producing hydroxyl radicals (oxidative potential +2.8 V). Meanwhile, the O_2_ molecule traps the conduction band electrons, generating a superoxide anion radical (^●^O_2_^−^). These reactive radicals decompose pollutants into less harmful substances in a quick and non-selective manner. The equations below illustrate the potential interfacial reactions that may occur [52,53,54,55].

NPs + light → h^+^ _(VB)_ + e^−^ _(CB)_ (4)

e^−^ _(CB)_ + O_2_ → radical ^●^O_2_^−^ (5)

h^+^ _(VB)_ + H_2_O → ^●^OH (6)

^●^O_2_^−^/^●^OH + substrate → degradable products (7)

An alternate possibility involves the oxidation of substrate molecules by positive holes (h+) which possess a high oxidative potential, as demonstrated by Equation (7) [56].

h^+^ _(VB)_ + substrate → substrate^+^ → degradable products (8)

**Figure 6:**
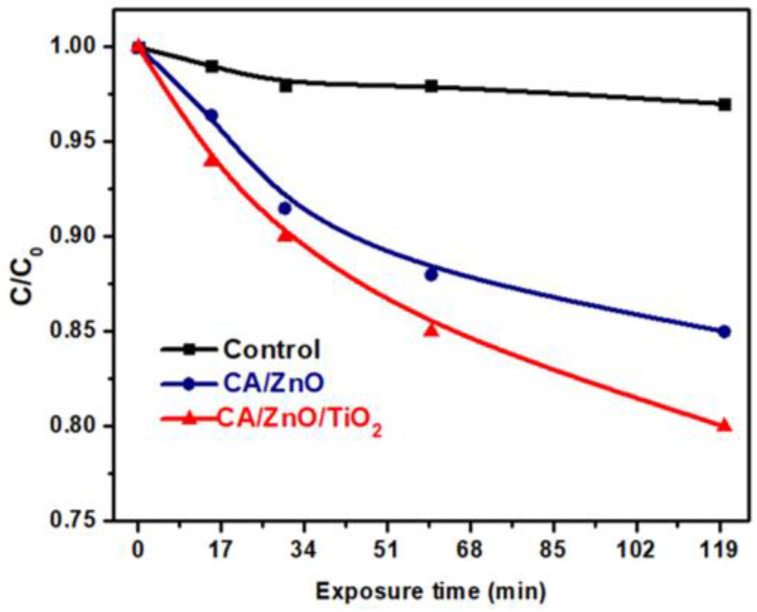
Membranes’ photocatalytic performance using MB dye. Control is referring to MB degradation without any catalyst materials.

**Figure 7:**
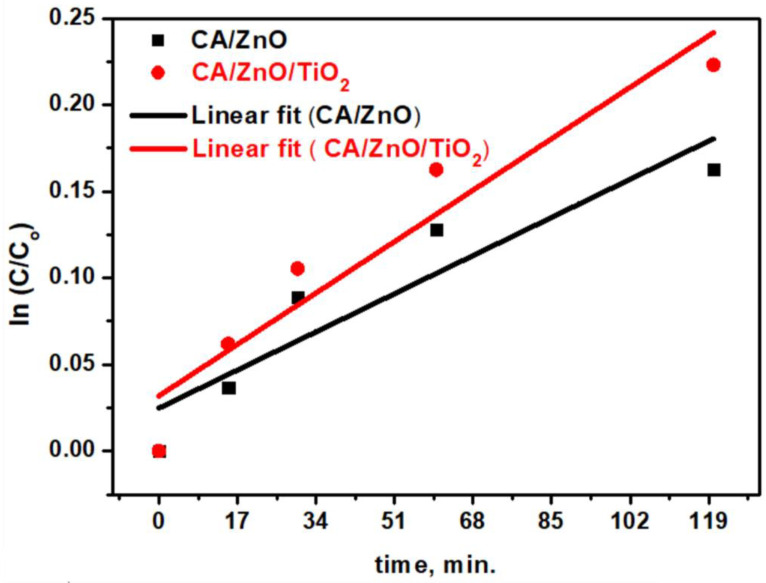
Plots of ln(C/C0) versus time for the prepared photocatalyst membrane against degradation of MB.

The results of MB photodegradation are introduced in Figure 6. The study examined the photodegradation curve with respect to the duration of light exposure (120 min) at different intervals of time. This was achieved by adding 15 cm^3^ of MB solution and photocatalytic membrane and exposing the solution to direct sunlight at different intervals of time. Subsequently, an evaluation was conducted on the CA/ZnO @ TiO_2_ NPs photocatalytic membranes using a UV spectrometer to measure absorbance at a wavelength of 64 nm for MB. The prepared composite membranes exhibit a remarkable capacity for photodegradation, which is sustained for 120 min under sunlight intensity. Irradiating the membrane commenced promptly after immersion in the solution, without any supplementary duration for soaking. The MB control exhibited little degradation, whereas the CA/ZnO @ TiO_2_ NPs membrane demonstrated absorption of 15% of MB after 30 min. After 120 min, about 20% of the MB dye was absorbed under sunlight. Figure 7 displays the photocatalytic degradation performance of organic dye using CA/ZnO and CA/ZnO/TiO_2_ materials as active photocatalyst substrates. The degradation points were found by estimating the exact content or molarity to the initial molarity against the estimated time of sunlight exposure. The data of dye photocatalytic degradation indicated the acceptable photodegradation ability of the prepared CA/ZnO and CA/ZnO/TiO_2_ with higher performance in the case of the CA/ZnO/TiO_2_ substrate, which could be due to the existence of a superior semiconductor in its chemistry (TiO_2_).

To describe the degradation kinetics of the utilized dye, the pseudo-first order was applied to fit the experimental data as shown in Figure 7:

Ln (C/C_o_) = kt (9)

The first-order rate constants for the prepared CA/ZnO and CA/ZnO/TiO_2_ materials were 0.0013 min^−1^ and 0.00175 min^−1^, respectively. Experimentally, the kinetics of dye degradation is faster by 34% in the case of the CA/ZnO/TiO_2_ material if compared with the CA/ZnO material. This could be attributed to the existence of Titania, which was reported to be the best semiconductor oxide up to now.

**Figure 8:**
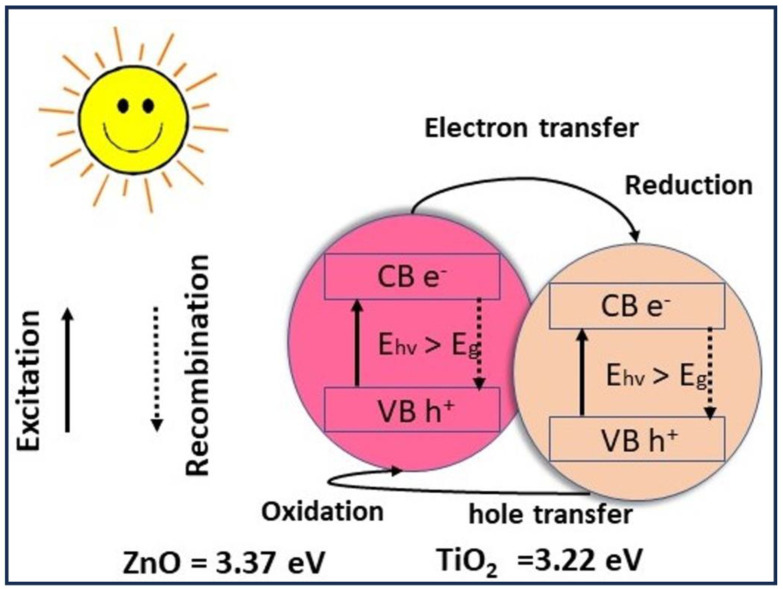
Photocatalytic process and electron transport of a composite CA/ZnO @ TiO_2_ membrane under direct sunlight.

Figure 8 is a schematic illustrating the composite photocatalytic mechanism when exposed to sunlight. Methylene blue (MB) was absorbed onto the surface of the photocatalyst. Many oxygen vacancies can be produced by the deposition of Ti^3+^ on the outer surface rather than Zn^2+^. The composite nanoparticles increased photocatalytic activity, which occurs due to electron transfer between TiO_2_ and ZnO. Consequently, the charge carriers produced after the absorption of visible-light radiation are subsequently on ZnO’s conduction band (CB) of 3.37 eV and TiO_2_’s valence band (VB) 3.22 eV, as reported in reference [57]. Yi Zhou et al. [49], by leveraging TiO_2_’s larger band, CB, were able to demonstrate electron transfer from the surface of the ZnO structure to the TiO_2_ one via the Z-scheme photocatalytic mechanism. Dye photodegradation is boosted as electrons transported to the TiO_2_ structure could recombine with the generated holes on the Titania VB. These electrons (h^+^) gather on the surface of ZnO and interact with oxygen molecules that have been absorbed to generate superoxide radicals (^•^O^2−^). The active chemicals (^•^O^2−^, h^+^) degraded MB into inert molecules.

**Figure 9:**
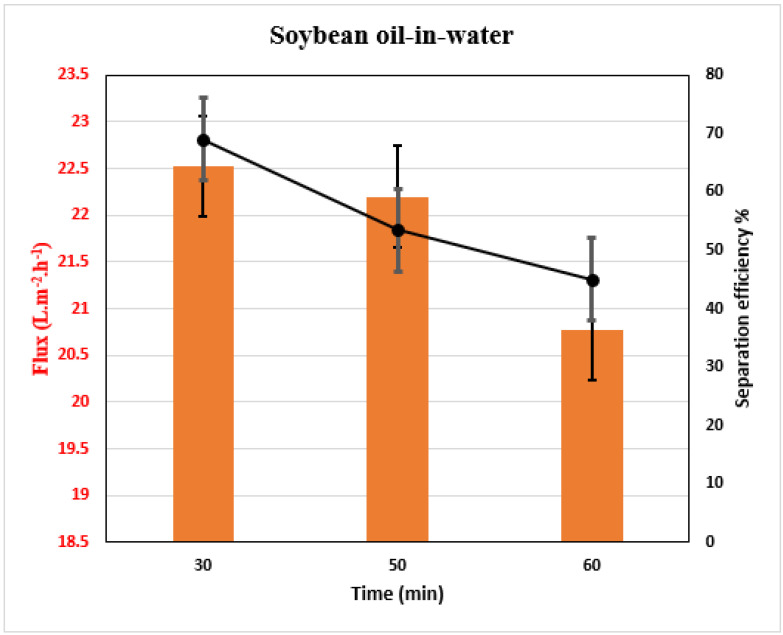
Membrane’s water flux and oil separation efficiency of modified CA/ZnO @ TiO_2_ NPs membrane.

The separation of the oil–water mixture is illustrated in Figure 9. The CA/ZnO @ TiO_2_ NPs membrane demonstrates excellent permeation flux and oil rejection during the separation period, followed by a slight decrease in water flux after a continuous operation of 1 h. The observed increased water flux can be due to various factors, including the porous network architecture of electrospun membranes and the hydrophilic nature of the membranes, primarily due to the chemical existence of ZnO beside TiO_2_ NPs. The network structure facilitates the transportation of water molecules across the membrane by providing additional pathways, thereby simplifying their transmission [58]. The CA/ZnO @ TiO_2_ NPs membrane keeps a final average water flux value of oil/water emulsion 22.5 L.m^−2^ h^−1^, which decreased to 20.7 L.m^−2^ h^−1^. Figure 9 displays the outcomes of the oil-in-water separation procedure, including the time-dependent permeation of membranes throughout the separation process. Furthermore, the membranes exhibit a remarkable oil rejection rate of 66%.

**Figure 10:**
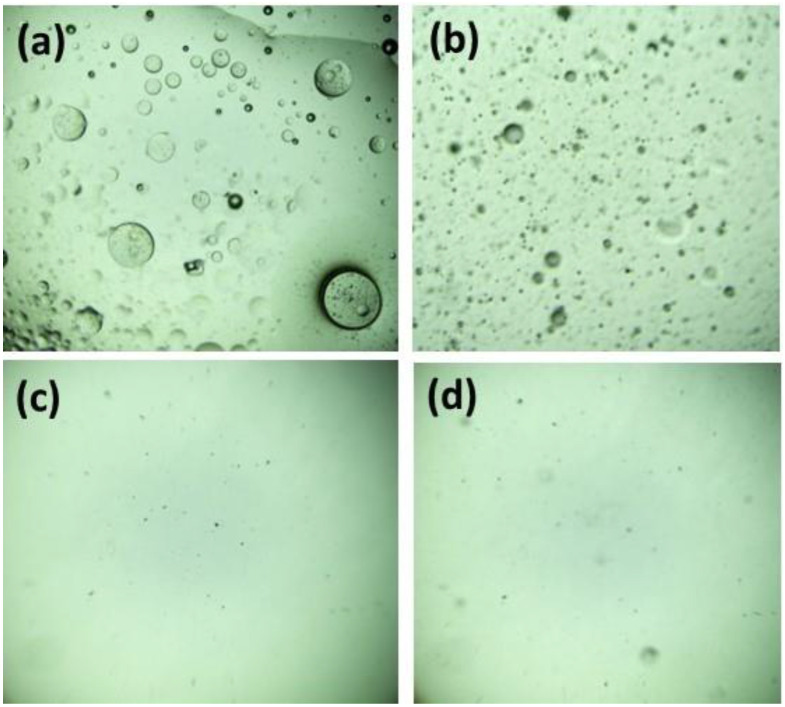
Optical microscope photographs of oily wastewater before and after separation were recorded for three distinct membranes. (**a**) oil/water emulsion before separation (**b**) CA, (**c**) CA/ZnO NPs, and (**d**) CA/ZnO @ TiO_2_ NPs. Microscopic images at a magnification of 10×.

Upon contact between the water-in-oil emulsion and membrane, the emulsion droplets broke apart onto the interface of the nanofiber’s membrane during the oil/water separation process. Subsequently, the aqueous component of the emulsion permeated the nanofiber’s membrane and was collected. The droplets present in a soybean-and-water emulsion and permeate were scrutinized using an optical microscope in order to validate the high separation efficacy of membranes for oil–water emulsion. Figure 10b–d display optical micrographs of the as-prepared water-in-oil emulsions before and after separation using the developed membranes. Several droplets were detected in the filtrate of the pristine CA membrane. In contrast, optical microscopy revealed no oil droplets in the filtrate produced by the CA/ZnO NPs and CA/ZnO @ TiO_2_ NPs membranes, demonstrating their high-efficiency oil–water separation capabilities.

## 4. Conclusions

A facile multifunctional TiO_2_-coated membrane was fabricated for utilization as a membrane for dye removal and gravity-driven oil–water separation. The membrane’s SEM image and contact angle measurement confirm the surface roughness and wettability necessary for efficient oil–water separation. The multifunction-coated membrane efficiently removed methylene dye from separated water due to the excellent photocatalytic properties of ZnO and TiO_2_ NPS when exposed to UV illumination. This is due to ZnO and TiO_2_ NPs being effective photocatalysts for the photocatalytic destruction of organic contaminants and microorganisms like sulfur-reducing bacteria found in crude oil. Furthermore, it was observed that the CA/ZnO @ TiO_2_ NPs membrane exhibited a significant separation efficiency of 45% during the oil–water separation process and an excellent water flux of 20.7 L.m^−2^ h^−1^ after 1 h. The aforementioned facts suggests that through appropriate enhancements in material synthesis, modifications to the photocatalytic process, and improvements to the oil-water separation system, the produced membrane exhibits the capacity to serve as a versatile membrane capable of concurrently facilitating the decomposition of organic pollutants within the water and separating oil from water. The current investigation contributes to enhancing cutting-edge solar irradiation technologies integrated with oil–water separation materials, which hold potential for commercial utilization.

## Data Availability

No new data were created in this study. Data sharing is not applicable to this article.
